# Cooperative management practices how to influence the productive performance outcomes: Based on the analysis of China’s guangxi 94 farmers professional cooperatives

**DOI:** 10.1371/journal.pone.0338545

**Published:** 2026-01-29

**Authors:** Ying-dong Zhao, Xin-chao Pan, Zi-lun Zhi, Chuan-yong Tang, Jie Dang, Kun-lin Bai

**Affiliations:** 1 School of Geography and Planning, Nanning Normal University, Nanning, Guangxi, China; 2 School of Primary Education, Nanning Normal University, Nanning, Guangxi, China; 3 Party Committee United Front Work Department, Nanning Normal University, Nanning, Guangxi, China; Yunnan University, CHINA

## Abstract

**Objectives:**

Research on the key factors that influence the productive outcomes of farmer professional cooperatives (FPCs) is essential to develop targeted management approaches that promote sustainable growth. This study investigates how cooperative management practices influence productive performance outcomes through an analysis of 94 FPCs in Guangxi, China.

**Methods:**

Employing cluster analysis, Ordinary Least Squares (OLS) regression, and mediation effect tests, the research categorizes management practices into five typologies (Comprehensive High-Efficiency, Collaboration-Deficient, Monitoring-Deficient, Planning-Deficient, Holistically Deficient) and links these to five distinct performance outcomes (Profitable Outreach High-Efficiency, Loss-Making Insular Low-Efficiency, etc.).

**Results:**

Results reveal that management practices critically determine performance outcomes types. Regression analysis highlights planning practices significantly influenced profitability, while investment practices positively impacted employment generation. Internal governance practices were strongly associated with institutional compliance, and external collaboration practices had a significant effect on technology dissemination and community reputation.Monitoring practices, while not directly impacting performance outcomes, were found to enhance them through management practices and mechanisms. Key behavioral indicators—revenue planning, capital investment, staff capacity, and role optimization—emerge as primary levers for performance optimization.

**Conclusion:**

This study underscore the critical role of management practices in shaping the performance of FPCs, suggestions include emphasizing efficient planning, investing in personnel training, and establishing dynamic adjustment mechanisms to ensure sustainable development. These findings provide empirical insights for optimizing rural collective governance and informing evidence-based policy interventions in developing agricultural economies.

## Introduction

As a significant economic organizational form, farmer professional cooperatives (FPCs) play an increasingly prominent role in promoting agricultural development, enhancing farmers’ incomes, and driving rural economic prosperity. The emergence and development of cooperatives represent an innovation and breakthrough from traditional agricultural production models. By organizing scattered farmers, these cooperatives facilitate resource sharing, risk diversification, and mutual benefit, thereby substantially improving agricultural productivity and advancing modern agricultural development (He & Chen, 2024) [1]. However, the growth trajectory of cooperatives has not been without challenges. Multiple operational obstacles persist during their development processes. Internally, the inherent complexity and diversity of cooperative management have rendered organizational efficiency a critical determinant of operational outcomes. Effective governance mechanisms can optimize resource allocation, enhance production efficiency, and reduce transaction costs, ultimately strengthening competitiveness and profitability (Saleh et al., 2024) [2]. Externally, Environmental dynamics and policy support levels exert a significant influence on the performance of cooperatives. Market volatility, competitive pressures, and policy uncertainties may collectively hinder development of cooperatives (Liu et al., 2023) [3]. Furthermore, output disparities exist among different cooperative types. For instance, production-oriented cooperatives and processing/marketing-focused cooperatives demonstrate distinct performance metrics and influencing factors (Li, 2019) [4].

This multidimensional complexity highlights the need to systematically identify key factors affecting productive outcomes of cooperatives. Understanding these determinants is crucial for developing targeted management approaches and designing evidence-based policies that foster sustainable growth.

Current research on the outcomes of cooperatives predominantly focuses on three thematic clusters: developmental trajectories, analysis of limiting factors, and corresponding countermeasure formulations. Scholars have identified multiple systemic constraints hindering the development of cooperatives, including small operational scale, inadequate internal governance structures, shortages of managerial expertise, insufficient capital-raising capacities, weak market competitiveness, excessive government dependency, deficient data tracking systems, and lack of internal/external oversight mechanisms (He & Chen, 2024; Kalogiannidis et al., 2024; Qu et al., 2023) [1,5,6]. In response, researchers have proposed multifaceted optimization strategies encompassing organizational standardization, institutional innovations, enhanced policy support and public awareness campaigns, alongside legal system improvements and government procurement mechanism reforms (Christian et al., 2024; Cui et al., 2016; Tang, 2019; Zhao & Liu, 2022) [7–10].

Existing scholarship predominantly examines productive outcomes of cooperatives through performance evaluation frameworks and analyses of external determinants such as policy environments and ecological factors. While these approaches effectively diagnose operational challenges and propose remedial measures, two critical gaps persist: (1) insufficient examination of endogenous behavioral determinants rooted in governance mechanisms, and (2) absence of taxonomical analysis differentiating developmental trajectories across cooperative typologies.

Addressing these knowledge gaps, this investigation establishes a novel analytical framework integrating management practices with productive outcomes. Employing a mixed-methods approach, we systematically analyze 94 farmer professional cooperatives (FPCs) in underdeveloped regions of Guangxi, China. The research design incorporates:

A hierarchical evaluation matrix quantifying 10 dimensions

Multivariate regression models identifying key performance predictors

Path analysis elucidating behavioral-outcome transmission mechanisms

This methodological reveals previously undocumented causal relationships between managerial decision-making patterns and cooperatives productivity. The findings advance theoretical understanding of FPCs growth dynamics while providing empirically grounded guidelines for:

Optimizing rural collective governance structures

Formulating differentiated development roadmaps

Enhancing policy intervention precision

This research showcases significant innovation in exploring the relationship between management practices and performance in Farmer Professional Cooperatives (FPCs). Specifically, it transcends the limitations of traditional single-dimensional performance evaluation (e.g., focusing solely on economic indicators) by proposing a multi-dimensional dynamic analysis framework that integrates management practices with performance outcomes. This framework encompasses multiple dimensions, including economy (profitability), institution (compliance), technology (technology dissemination), and society (employment, community reputation). Furthermore, the study categorizes management practices into five behavioral dimensions: planning, investment, internal governance, external collaboration, and monitoring. By doing so, it elucidates the dynamic correlation between multi-dimensional management behaviors and composite performance results, establishing a mapping relationship between “management practices” and “performance outcomes.” This provides a systematic analytical tool for advancing the study of FPCs.

## Theoretical analysis and research hypothesis

### The impact of management practices on outcomes of FPCs

Management practices play a pivotal role in enhancing productive performance outcomes of FPCs. Management practices encompass decision-making processes, resource allocation strategies, operational planning frameworks, and performance monitoring systems—interconnected dimensions that dynamically interact through reciprocal causation mechanisms. Proactive decision-making protocols enable FPCs to swiftly adapt to market fluctuations and internal operational demands, thereby minimizing inefficiencies and capitalizing on revenue-generating opportunities (Zhou et al., 2020) [11]. Empirical research confirms that strategic resource allocation mechanisms—especially those enabling effective workforce deployment and competency-focused role specialization—significantly enhance organizational efficiency (Tran et al., 2023) [12]. Methodical planning frameworks, incorporating rigorous budget design and capital utilization oversight, not only safeguard financial sustainability but also catalyze production scale expansion through technological modernization (Chiranjeewee et al., 2024; Zhao et al., 2024) [13,14]. Furthermore, integrated monitoring systems incorporating real-time market analytics, cross-industry technological bench-marking, and operational performance diagnostics play a pivotal role in facilitating data-driven strategic decisions and enabling continuous process optimization (Vitalii & Liu, 2022) [15]. In summary, FPCs that achieve superior productive outcomes in competitive market landscapes demonstrate systematic optimization of managerial processes across operational stages, particularly in decision-making agility and resource allocation efficiency.

Concurrently, productive performance outcomes of FPCs extends beyond mere operational profitability. It encompasses a broader spectrum, including the standardization of the cooperative's organizational structure, the engagement levels of participating entities, the satisfaction of stakeholders, and the social impact generated by the projects undertaken. In essence, it represents a synthesis of process-oriented and outcome-based performance (Jamaluddin et al., 2023; Qiao et al., 2024) [16,17]. Against this backdrop, the management practices of FPCs across various domains, such as decision-making, resource allocation, strategic planning, and information-monitoring, exert distinct influences on the diverse facets of their performance outcomes. However, at present, the academic circle still lacks research on how FPCs management practices affect their performance outcomes.

Building upon the preceding analysis, this study proposes the following research Hypothesis H1: The diverse management practices of FPCs are capable of enhancing varied productive performance outcomes.

### Outcomes types of FPCs

Existing typologies delineate FPCs along multiple institutional dimensions. Product-based categorization identifies three primary clusters: agro-product cooperatives, artisanal collectives, and service-oriented entities (Qorri & Felföldi, 2024) [18]. Ownership governance paradigms differentiate between indigenous cooperatives, corporate-affiliated hybrids, and enterprise-dominant models (Wan & Zeng, 2020) [19]. Genes drivers bifurcate cooperatives into farmer-initiated versus state-sponsored archetypes (Wang et al., 2021) [20]. Property-rights configurations yield four distinct governance modes: equity-concentrated, democratic-ownership, decision-centralized, and patron-contributory structures (Lu et al., 2018) [21]. Institutional sponsorship matrices further classify cooperatives into community-based, agro-processing enterprise-linked, government-guided, specialized household-led, and supply-and-marketing cooperative derivatives (Wu, 2013) [22]. Additionally, tripartite entitlement configurations (ownership-control-benefit) delineate traditional, transitional, and modernized cooperatives (Liao, 2013) [23]. While these studies systematically examine formative drivers and institutional barriers shaping cooperative hypogenesis, a critical analytical gap persists: the absence of performance-based taxonomies accounting for productive performance outcomes differentials.

Consequently, it is feasible to categorize FPCs in accordance with the variations in the diverse dimensions of performance outcomes. This approach enables the identification of which management practices influence the type of outcomes, as well as the determination of the dominant management practices that lead to the differences in the types of cooperative outcomes. Drawing on the aforementioned analysis, this study posits the following research Hypothesis H2: Management practices characteristics determine the type of performance outcomes of FPCs.

## Research design

### Data sources

The datasets originates from a comprehensive evaluation program conducted by the authors across 10 counties (districts) in underdeveloped regions of Guangxi Province. It includes 94 Farmer Professional Cooperatives (FPCs). These cooperatives cover a range of key sectors—including crop farming, animal husbandry, agricultural processing, and agricultural services—thereby reflecting the primary industries in which agricultural cooperatives typically operate. Furthermore, all FPCs are located in less developed areas of Guangxi, making their developmental contexts representative of those found in many underdeveloped regions in China. A stratified random sampling approach was employed, with initial outreach to 126 registered FPCs for longitudinal tracking between 2020 and 2024. Performance metrics were systematically captured through:

Semi-structured interviews with board chairs (n=126)

Administrator-completed standardized questionnaires (effective ratio: 98.4%)

Archival review of operational records and financial statements

Our multi-wave assessment protocol measured both developmental progress (assessed via practices maturity indices) and outcomes (assessed via outcomes value-added indices). However, terminal-phase verification in 2024 identified 32 FPCs as non-operational entities, rendering key performance indicators (KPIs) including operational revenue and employment multipliers un-quantifiable. The productive performance score of these non-operating FPCs is 0, which needs to be excluded to avoid interfering with the regression results. Consequently, the analytical sample was refined to 94 actively functioning FPCs meeting the following viability criteria:

Continuous operation ≥2 fiscal years

Minimum member participation ≥30 households

Audit able financial reporting

### Variable selection

#### 1) Independent variable.

In this study, the core explanatory variable is management practices. Drawing on the research by Wu and Zhu (2018) [24], management practices is conceptualized as five distinct dimensions: planned practices, investment practices, internal governance practices, external collaboration practices, and monitoring practices. These five dimensions are designated as independent variables. Subsequently, each of these practices is further subdivided into specific behavioral indicators, and the scores of these indicators are assessed. This approach allows for a more detailed and comprehensive analysis of the various dimensions of management practices and their respective impacts.

As detailed in Table 1, each dimension is decomposed into 3 measurable behavioral indicators. Practical intensity is quantified through a validated 4-tier scale:

**Table 1:** Management practices evaluation indicator system.

Variable type	Variable name	Evaluation indicator	Measurement criteria: 4-level scale
Independent variable	Planned practices	Business plan	3 = A detailed business plan has been formulated with good implementation status. 2 = A detailed business plan has been formulated; however, problems were encountered during its implementation, necessitating adjustments to the plan. 1 = A detailed business plan has been formulated, but it has only just been implemented and no problems have been identified thus far. 0 = A preliminary business plan has been formulated, but it has not yet been implemented.
		Financial plan	3 = A detailed financial plan has been formulated with clear understanding of fund utilization. 2 = A detailed financial plan has been formulated, but adjustments are still needed in the use of funds. 1 = A detailed financial plan has been formulated, but it has only just been implemented and no problems have been identified yet. 0 = A preliminary financial plan has been formulated, but it has not yet been implemented.
		Revenue sources plan	3 = Pre-planned revenue sources with stable income streams. 2 = The revenue sources were pre-planned, but the actual income fluctuated greatly. 1 = The revenue sources were pre-planned,but the market changes greatly and it is necessary to adjust the sources of income. 0 = The general revenue sources were pre-planned, but the implementation has not yet begun.
	Investment practices	Capital investment intensity	3 = Significant investment and diversified financing channels to resolve funding issues. 2 = A large amount of funds has been invested, but the gap remains significant. 1 = After investing the basic funds, it was found that a large number of resources were still needed to carry out the operation and could not be solved. 0 = The basic funding has been invested but the operation is not yet formal
		Facility & equipment	3 = Full production capacity with complete equipment setup. 2 = It has been in operation and still lacks equipment to expand production. 1 = Lack of some equipment, can only maintain basic operations. 0 = At present, key equipment is still lacking, making it impossible to carry out business operations.
		Staff capacity investment	3 = Managers received various competency training. 2 = Managers have undergone competency training; however, there are still some operational issues that are proving difficult to resolve. 1 = Managers received short-term training that yielded limited results. 0 = Managers have not received competency training.
	Internal governance practices	Record keeping rigor	3 = Maintained complete records of production/sales/expenses/meetings. 2 = Only the important production/sales records were retained. 1 = A small number of scattered records. 0 = No records were retained.
		Staff allocation optimization	3 = Clear division of responsibilities and supervision of implementation. 2 = Clear division of responsibilities, but there is no supervision. 1 = There is a fundamental division of responsibilities, but the effectiveness of this division is poor. 0 = There is no division of responsibilities, it is decided by the chairman.
		Conflict resolution	3 = Resolve disputes through an internal conflict plan. 2 = Addressed disputes according to village committee procedures. 1 = Conflicts are resolved through internal consultations, but not in a standardized manner. 0 = No conflict, no plan.
	External collaboration practices	Stakeholder collaboration depth	3 = Official collaborated with enterprises in the industry chain. 2 = Only verbal cooperation has been reached with the enterprises in the industry chain. 1 = There have been consultations, but it was found that there are very few opportunities for cooperation. 0 = Not considered this issue.
		Relationship management	3 = Engaged in joint sales with other FPCs. 2 = Communicate market information with other FPCs regularly. 1 = Communicated with others FPCs, but find it is of little help to oneself. 0 = Not considered this issue.
		Community partnership	3 = Conducted transactions and cooperation with neighboring villagers. 2 = There are regular transactions with the surrounding villagers, though they are very limited. 1 = Upon investigation, it was determined that there was no correlation between the surrounding villagers and the cooperative's products. 0 = Not considered this issue.
	Monitoring practices	Assess own advantages	3 = Regularly assess one's own advantages within the industry operation, and there are indeed advantages. 2 = Upon regularly comparing the industry, it becomes evident that there are no advantages. 1 = Upon finding the industry saturated, consider changing industries. 0 = Not considered this issue.
		Market information utilization	3 = Proactively explored markets and gathered market intelligence. 2 = Market information has been collected multiple times, yet it has not been fully utilized. 1 = Only a limited number of channels are used to collect market information, and the information gathered is not comprehensive. 0 = Not considered this issue.
		Performance monitoring	3 = Regularly evaluated production/sales performance and analyzed existing issues. 2 = Production and sales information is collected regularly, but it is not analyzed. 1 = Collecting a small amount of production and sales information is not useful. 0 = Not considered this issue.

3 = Full compliance with best practice benchmarks

2 = Partial compliance with identifiable issues

1 = Action implemented but with insignificant outcomes

0 = Non-compliance or unknown

The management practices evaluation framework adopted in this study originates from the protected area management assessment field, having been jointly developed by the World Bank and World Wide Fund for Nature (WWF). Originally designed for systematic monitoring and evaluation of protected area development, this methodology represents a well-established scientific measurement approach characterized by rigorous design and systematic control (Hockings, 2003) [25].

A key advantage of this methodological approach lies in its ability to assess a wide range of non-quantifiable indicators, rendering it particularly suitable for evaluating management practices. The assessment protocol incorporates a dual-verification system that combines structured interviews with statistical documentation to mitigate subjective bias. It employs a four-tier classification framework, with clearly defined scoring criteria for each level, thereby minimizing evaluative ambiguity and promoting consistent, evidence-based judgments. This design enables the systematic transformation of qualitative observations into quantifiable and comparable discrete data. Additionally, all data collection is conducted under standardized conditions using established protocols to reduce potential contamination from external factors such as time constraints or environmental interference. As a result, the datasets generated supports robust quantitative analysis of management behaviors and fully satisfies the parametric assumptions required for subsequent statistical modeling.

The variables are measured through a structured scoring procedure based on interviews with the FPC’s management team and a review of supporting documentation. During the assessment of the action plan, management representatives are asked to describe the formulation and implementation of the plan, supplemented by relevant written materials. Scoring is then conducted according to the degree of plan completion, using a 4-level scale.

#### 2) Dependent variable.

The dependent variable in this study—productive performance outcomes—is ope-rationalized through a multidimensional evaluation framework encompassing economic, environmental, and social dimensions (Barry, 2021; Marcis et al., 2019; Nguyen et al., 2024; Sebhatu et al., 2021; Zeng et al., 2024) [26,27–30]. We refine this construct into five measurable components:

Profitability (economic efficacy): assessed via financial statement analysis to quantify revenue streams and net gains.

Institutional compliance (governance standardization): evaluated through audits of ledgers, equity distribution records, and structured interviews to detect malpractices (e.g., asset misappropriation or reporting irregularities).

Technology dissemination: measured by member surveys and neighboring villager interviews to gauge adoption rates of agricultural innovations.

Employment generation: calculated using full-time equivalent metrics derived from payroll records and employment contracts.

Community reputation: quantified through sentiment analysis of villager narratives regarding cooperative legitimacy and social impact.

Adopting a composite scoring methodology, this framework enables quantification of both tangible and intangible outcomes (e.g., institutional compliance, technology dissemination). Mirroring the management practices metric system (Table 1), performance outcomes indicators are scored on a validated 4-tier scale:

3 = Exemplary compliance with sector benchmarks

2 = Functional implementation with correctable deficiencies

1 = Incipient efforts lacking measurable outcomes

0 = Non-implementation or undocumented actions

As detailed in Table 2.

**Table 2:** Productive performance outcomes evaluation indicator system.

Variable type	Variable name	Measurement criteria: 4-tier scale
Dependent variable	Profitability	3 = Net profit is positive 2 = EBITDA (Earnings Before Interest, Taxes, Depreciation, and Amortization) is positive 1 = Has operating income, EBITDA is negative 0 = No operating income
	Institutional compliance	3 = Financial regulations, public disclosure of board personnel, clear membership shareholding, democratic decision-making 2 = Many financial issues, problems with member share capital, decisions not subject to democratic oversight 1 = Personnel incomplete, share capital contributions not fully paid, no trustee personnel 0 = Not officially operational
	Technology dissemination	3 = Villagers around also receive training from the cooperative and use it for production 2 = Members receive training from the cooperative and use it for production 1 = The cooperative has organized some technical training, but the results are not good 0 = No technical training
	Employment generation	3 = Employing 10 or more workers, annual labor costs exceed 200,000 *yuan* 2 = Employing 10 or more workers, annual labor costs range from 50,000–200,000 *yuan* 1 = Employing 2–10 workers, annual labor costs are less than 50,000 *yuan* 0 = Employing fewer than 2 workers, annual labor costs are less than 5,000 *yuan*
	Community reputation	3 = Villagers comprehend the cooperative, all reviews are positive 2 = Villagers comprehend the cooperative, but a few do not recognize it 1 = Villagers do not comprehend the cooperative, yet recognize the cooperative management personnel 0 = Villagers neither comprehend the cooperative nor are aware of the cooperative management personnel

#### 3) Control variables.

Drawing on the methodological framework established by Shao et al. (2014) [31] and extended by He et al. (2024) [32], this study incorporates seven control variables to mitigate confounding biases in regression analyses:

Operational tenure (years since establishment)

Membership size (total registered members)

Leadership-political alignment (board chairs holding village committee roles)

Geo-spatial clustering (administrative district classification)

Sectoral typology (primary industry categorization: agriculture/artisan/services)

External fiscal subsidies (government grants or donor funding)

Differences in managerial competence (educational background of the Chairman)

The selection of these control variables is used to enhance the accuracy of regression analysis.

### Selection of analysis method and model

#### 1) Cluster analysis.

This study employed the K-Means clustering algorithm via the SPSSPRO statistical analytics platform to conduct typological segmentation of FPCs outcome patterns, thereby indirectly validating hypothesis H2. The K-Means algorithm operates on the principle of iteratively optimizing K centroid positions to minimize intra-cluster variance while maximizing inter-cluster dissimilarity. Methodologically, we preprocessed the datasets by excluding 32 non-operational FPCs, retaining 94 active entities for analysis. The optimal cluster count was determined to be K = 5 using the elbow method, which validated a reduction in SSE (sum of squared errors) of less than 5% beyond K = 5. And executed multiple iterations with k-means initialization to avoid sub optimal convergence.

The clustering framework simultaneously analyzed management practices and productive performance outcomes, encompassing 5 management dimensions (Table 1), and 5 outcome indicators (Table 2).

Cluster robustness has been tested. SPSSPRO was selected for its validated implementation of machine learning pipelines, which aligns with the requirements for mid-sized cohort analysis in agricultural economics research.

#### 2) Profiling analysis.

Profiling analysis, a methodological approach traditionally employed in market segmentation and product innovation through systematic data pattern recognition, is adapted in this study to characterize the nexus between management practices and productive performance outcomes clusters. Leveraging K-Means-derived typologies, we conducted multidimensional feature extraction using parametric statistical descriptors (mean) to construct archetypal profiles for each FPCs cluster.

The resultant unified data schema achieves discriminative accuracy in cluster differentiation, thereby providing empirical substantiation for Hypothesis H2 regarding management-outcomes relationships.

#### 3) Regression analysis.

This study employs a multivariate regression econometric model to investigate the relationship between independent variables (management practices) and dependent variable (productive performance outcomes). The formal specification is expressed as:


$${\text{Y}}_{\text{i}}=\text{c}+\alpha_{\text{i}}\;{\text{X}}_{\text{i}}+\beta_{\text{i}}\;\text{Control}+\mu \;\left(𝐌𝐨𝐝𝐞𝐥\;1\right)$$

Where:

Y: Performance outcomes dimensions (dependent variable)

X: Management practices dimensions (independent variables)

Control: Covariates (operational tenure, membership size, leadership-political alignment, geospatial clustering, sectoral typology, external fiscal subsidies)

c: Intercept term

α: Regression coefficients

β: Control variable coefficients

μ: Stochastic error term

The model evaluates which performance dimensions (profitability, compliance, etc.) are most responsive to management practices. Given significant multicollinearity between management practices indicators, we implement ridge regression—a regularized least squares estimator—to enhance model robustness. Methodologically, ridge regression enables rigorous testing of Hypothesis H1 by addressing inherent data limitations while preserving causal interpretability.

#### 4) Mediation effect test.

To further investigate the mechanisms through which management practices influence FPCs performance outcomes, this study employs a three-stage mediation analysis framework building upon baseline regression results. The mediation models are specified as follows:

Stage 1 (Direct effect):


$$\text{Y}=\text{cX}\;+\gamma_{\text{1}}\text{Control}\;+\in_{\text{1}}(𝐌𝐨𝐝𝐞𝐥\;2)$$

Stage 2 (Mediation pathway):


$$\text{M}=\text{aX}+\gamma_{\text{2}}\text{Control}\;+\in_{\text{2}}(𝐌𝐨𝐝𝐞𝐥\;3)$$

Stage 3 (Full mediation):


$$\text{Y}=\text{c}’\;\text{X}+\text{bM}+\gamma_{\text{3}}\text{Control}\;+\in_{\text{3}}(𝐌𝐨𝐝𝐞𝐥\;4)$$

Where:

Y: Performance outcomes dimensions (dependent variable)

X: Management practices dimensions (independent variable)

M: Mediator variables

Control: Covariates (operational tenure, membership size, etc.)

c,c′,a,b: Path coefficients

γ_1−3_: Control variable coefficients

∊_1−3_: Stochastic error terms

Mediation effects are determined through sequential examination of path coefficients:

Complete mediation: Established when coefficients a (X → M) and b (M → Y) are statistically significant (p < 0.05), while c′ (direct X → Y) becomes nonsignificant (p ＞ 0.05).
Partial mediation: Confirmed if a, b, and c′ are significant with a × b and c′ sharing directional consistency (i.e., same sign).
Suppression effect: Identified when a × b and c′ exhibit opposing signs despite significance.

To ensure robustness, we implement bootstrap resampling to derive bias-corrected 95% confidence intervals (CIs) for indirect effects (a × b). Mediation is validated if CIs exclude zero. This method is primarily used to test for indirect factors affecting outcomes in management practices, to verify hypothesis H1.

#### 5) Correlation analysis.

This study conducted Pearson correlation analysis using SPSSPRO to examine the associations between practices indicators and performance outcomes. The methodology specifically identifies which practices exert the most substantial influence on outcomes, with correlation magnitudes serving to validate Hypothesis H1 regarding behavioral determinants of performance differentiation.

## Results and analysis

### Descriptive statistics

**Fig 1:**
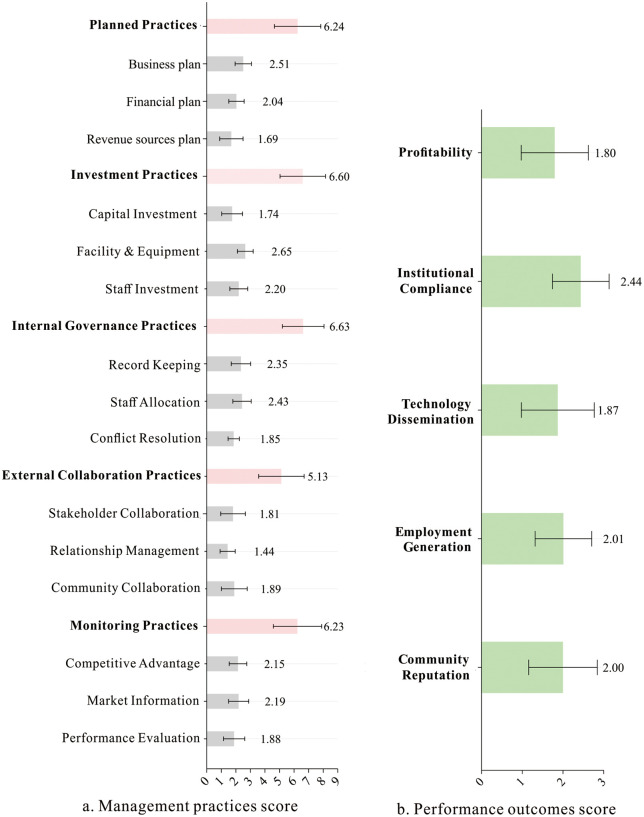
Variable feature statistics.

The descriptive statistics of key variables are presented in Fig 1. The results indicate that both management practices and productive performance outcomes exhibit moderate dispersion, with relatively balanced distributions approximating normality. This distributional property satisfies the parametric assumptions for subsequent cluster analysis, linear regression, and related statistical modeling.

### Testing of validity and reliability

First, a reliability analysis of the survey results was conducted. The Cronbach’s α coefficient for the five management practice variables (independent variables) was 0.91, and that for the five performance outcome variables (dependent variables) was 0.77. These values indicate that the data from both surveys were reliable and stable. Additionally, validity tests were performed during the survey process. For the five management practice variables (independent variables), the KMO test value was 0.863, and it passed the Bartlett test (P < 0.01), demonstrating significance and suitability for factor analysis. For the five performance outcome variables (dependent variables), the KMO value was 0.704, and it also passed the Bartlett test (P < 0.01), indicating significance and suitability for factor analysis. Consequently, the survey results can serve as a valid basis for further phenomenon analysis.

### Results and analysis

#### 1) Cluster analysis results.

**Fig 2:**
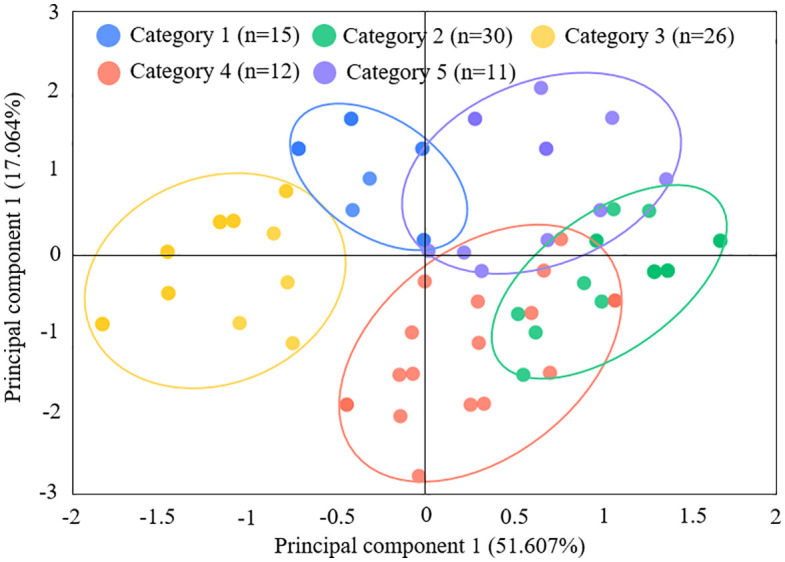
Performance outcomes clustering of FPCs.

Cluster analysis of FPCs productive performance outcomes (dependent variables) revealed five distinct clusters, with statistically significant differences among the groups (p < 0.001), validating the robustness of the classification. The distinct clustering patterns, characterized by variations in profitability, institutional compliance, and social impact metrics, are detailed in Fig 2.

**Fig 3:**
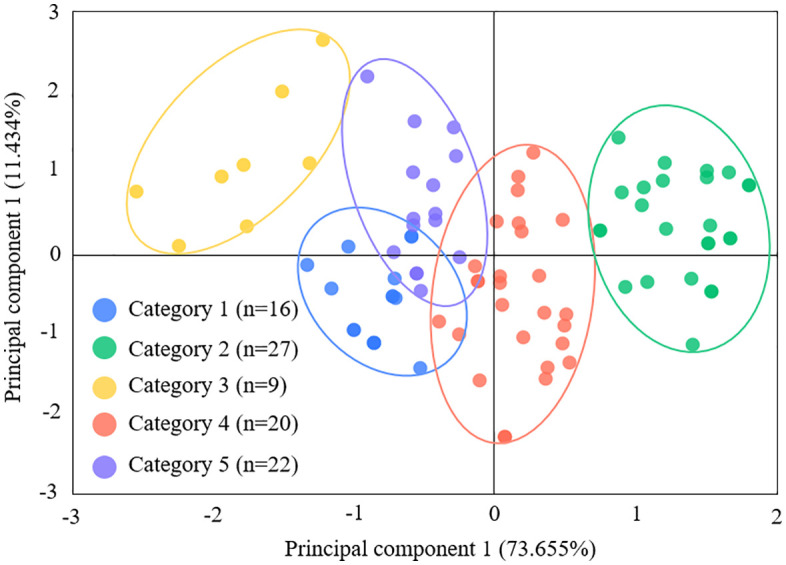
Management practices clustering of FPCs.

Similarly, cluster analysis applied to management practices (independent variables) identified five typologies, demonstrating significant inter-cluster divergence (p < 0.001). These practical archetypes, which are distinguished by their approach to planning, resource investment, governance, collaboration, and monitoring, are depicted in Fig 3.

#### 2) Type characteristic portrait.

**Fig 4:**
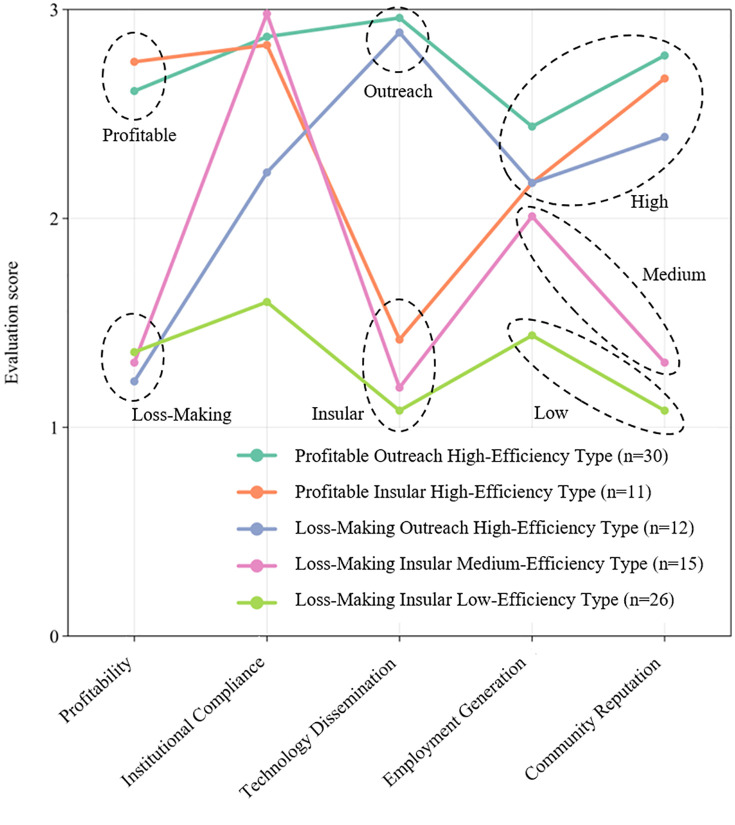
Portrait of performance outcomes types of FPCs.

Cluster analysis delineated five typologies of productive performance outcomes, each exhibiting distinct operational profiles (Fig 4):

Loss-Making Insular Medium-Efficiency Type (n = 15): poor technology dissemination (mean = 1.18 ± 0.40), negative profitability (mean = 1.23 ± 0.46), subpar community reputation (mean = 1.27 ± 0.41), yet high institutional compliance (mean = 2.95 ± 0.46). Its portrait features are financially unsustainable but managerially rigorous.
Profitable Outreach High-Efficiency Type (n = 30): exemplary performance across metrics—robust profitability (mean = 2.64 ± 0.41), advanced technology diffusion (mean = 2.91 ± 0.36), community endorsement (mean = 2.79 ± 0.36). Its portrait features are holistic optimization model.
Loss-Making Insular Low-Efficiency Type (n = 26): systemic deficiencies—chronic losses (mean = 1.27 ± 0.41), negligible technology transfer (mean = 1.08 ± 0.41), weak institutional governance (mean = 1.55 ± 0.51). Its portrait features are multi-dimensional underperformance.
Loss-Making Outreach High-Efficiency Type (n = 12): paradoxical combination—strong technology diffusion (mean = 2.83 ± 0.46) coexisting with financial deficits (mean = 1.18 ± 0.50), moderate compliance (mean = 2.22 ± 0.68). Its portrait features are socially impactful yet economically unviable.
Profitable Insular High-Efficiency Type (n = 11): profit-driven isolationism—high returns (mean = 2.81 ± 0.49) with limited technology sharing (mean = 1.46 ± 0.28), above-average compliance (mean = 2.83 ± 0.45). Its portrait features are financially successful but socially constrained.

**Fig 5:**
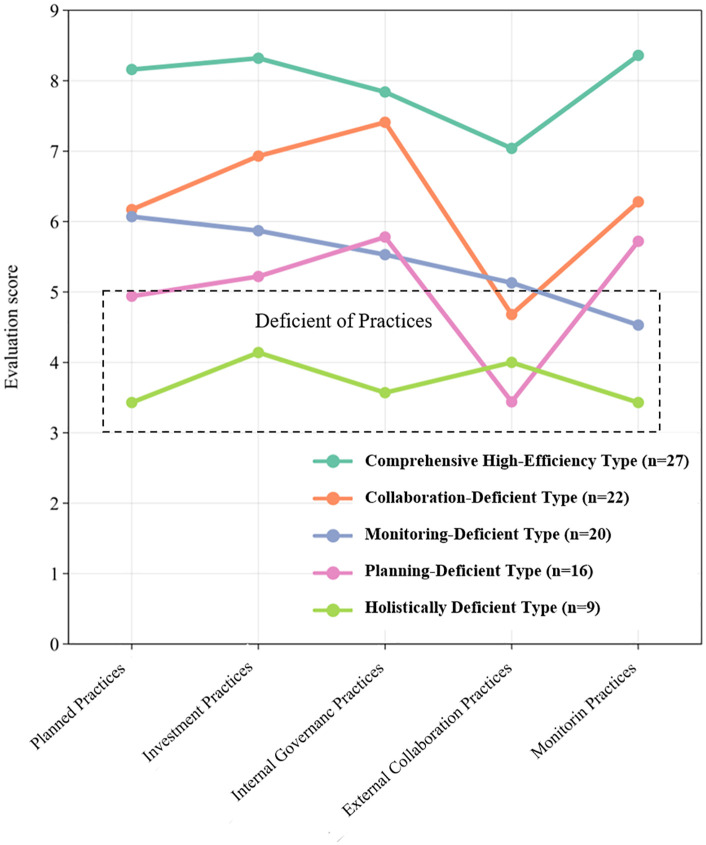
Portrait of management practices types of FPCs.

The management practices of FPCs were clustered into five distinct typologies, with a performance threshold of 5 points demarcating practice adequacy (scores <5 indicating deficiencies in respective practices). The typological profiles are characterized as follows (Fig 5):

Comprehensive High-Efficiency Type (n = 27): exemplary high scores across all management dimensions, particularly in planned practices (mean = 8.16 ± 0.85) and external collaboration practices (mean = 7.04 ± 0.84).
Collaboration-Deficient Type (n = 22): suboptimal external collaboration practices (mean = 4.82 ± 1.02) despite adequate internal governance practices (mean = 7.393 ± 0.68).
Monitoring-Deficient Type (n = 20): critical gaps in monitoring practices (mean = 4.67 ± 0.72), though maintaining external collaboration practices (mean = 5.13 ± 0.83).
Planning-Deficient Type (n = 16): weak planned practices (mean = 4.94 ± 0.54) and external collaboration practices (mean = 3.47 ± 0.87) coupled with stronger monitoring practices (mean = 5.72 ± 0.46).
Holistically Deficient Type (n = 9): systemic underperformance across all practices, indicating institutional fragility.

#### 3) Type association.

**Fig 6:**
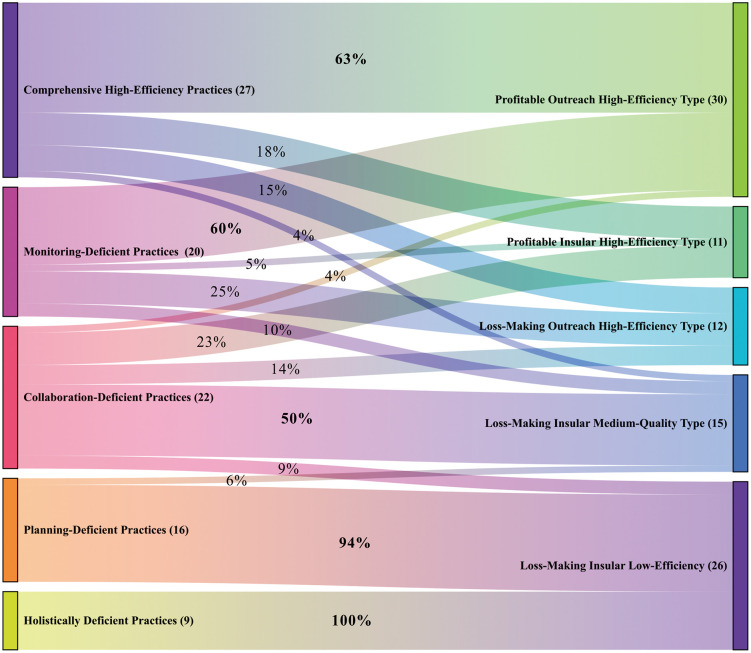
The association of performance outcomes with management practices.

The comparative analysis between management practices clusters and performance outcomes clusters (Fig 6) reveals significant inter-dependencies, demonstrating that management practices profoundly shape performance outcomes:

Comprehensive high-efficiency practices association (n = 27): 63% align with Profitable Outreach High-Efficiency, 18% with Profitable Insular High-Efficiency, and 15% with Loss-Making Outreach High-Efficiency. This demonstrates that holistic management ensures high performance despite minor trade-offs between profitability and technology diffusion.
Collaboration-deficient practices association (n = 22): 50% align with Loss-Making Insular Medium-Quality, 23% with Profitable Insular High-Efficiency, and 14% with Loss-Making Outreach High-Efficiency. This demonstrates that external collaboration deficits degrade outcomes quality, profitability unsustainability even among high-Efficiency clusters.
Monitoring-deficient practices association (n = 20): 60% align with Profitable Outreach High-Efficiency, while 25% become Loss-Making Outreach High-Efficiency. This demonstrates that monitoring mechanisms correlate with high-efficiency but mask profitability risks.
Planning-deficient practices association (n = 16): 94% align with Loss-Making Insular Low-Efficiency. This demonstrates that strategic plan is a critical determinant—its absence catastrophically reduces outcomes quality.
Holistically deficient practices association (n = 9): 100% align with Loss-Making Insular Low-Efficiency. This demonstrates that systemic governance failures ensure multi-dimensional underperformance.

The empirical analysis substantiates Hypothesis H2, confirming that management practices characteristics critically determine FPCs outcomes typologies. Institutional outcomes systematically degrade under specific governance deficiencies:

The absence of planning protocols leads to significant deterioration in outcomes, eroding both profitability and institutional efficiency.
Conversely, FPCs lacking monitoring mechanisms surprisingly maintain growth trajectories, achieving high outcomes quality albeit with profitable risks.
Restricted external engagement impedes technology diffusion and decelerates developmental momentum, yet preserves mid outcomes quality.

#### 4) Benchmark regression results and analysis.

To address the impact of multicollinearity on regression results, this study first conducted Variance Inflation Factor (VIF) tests on all variables. The findings revealed that the maximum VIF value reached 3.78, while the minimum was 1.93 with a mean of 2.51—significantly below the 5 threshold for detecting severe multicollinearity. These statistical results confirm that the model is free from significant multicollinearity issues. The Ordinary Least Squares (OLS) regression results, presented in Table 3, demonstrate that management practices exhibit a statistically significant positive impact on productive performance outcomes at the 5% significance level.

**Table 3:** Regression analysis of management practices and productive performance outcomes.

Variables	Productive Performance Outcomes Model 1 (OLS regression)				
	Profitability	Institutional compliance	Technology dissemination	Employment generation	Community reputation
Planning practices	0.263*** (0.078)				
Investment practices				0.169 ** (0.063)	
Internal governance practices		0.181 ** (0.071)			
External collaboration practices			0.392 *** (0.077)		0.37 *** (0.057)
Monitoring practices					
Control variables (Operational tenure, Membership size, Leadership-political alignment, Geospatial clustering, Sectoral typology, External fiscal subsidies, Differences in managerial competence)	Control	Control	Control	Control	Control
N	94	94	94	94	94
R^2^	0.677	0.608	0.607	0.662	0.76

***, ** and * represent significance levels of 1%, 5% and 10% respectively.

Notably, planning practices exerts exceptionally strong effects on profitability, with significance at the 1% level (p < 0.01). This indicates a dose-response relationship—enhanced investment in strategic planning correlates with increased operational expertise, driving financial returns.

Investment practices exhibited statistically significant positive effects on employment generation (p < 0.05), indicating that enhanced capital investment not only expands FPCs scale but also significantly increases workforce recruitment, thereby driving local employment. Furthermore, investments in personnel training were found to elevate managerial standardization, reinforcing the critical role of human capital development.

Internal governance practices demonstrated a positive association with institutional compliance (p < 0.01). These results underscore that systematic internal governance mechanisms enhance standardization and stable development.

External collaboration practices demonstrated statistically significant positive effects on technology dissemination (p < 0.01) and community reputation (p < 0.01), highlighting exchange and cooperation pivotal role in enhancing technical outreach and local evaluation.

Monitoring practices did not show a direct significant impact on productive performance outcomes (p > 0.10 across all metrics). However, typological analysis revealed that FPCs lacking robust monitoring practices were often clustered into high-risk outcomes categories, implying potential indirect mediation pathways. To disentangle these effects, we conducted a mediation analysis to examine whether monitoring practices influence outcomes indirectly.

This analysis extends FPCs governance theory by outlining the mechanistic pathways through which managerial practices synergetically improve outcomes.

#### 5) Robustness test.

To enhance the reliability and validity of the findings, robustness checks were performed on the benchmark regression results by modifying variable specifications and incorporating additional controls. Specifically, the following adjustments were made: (1) the measurement of the independent variable (management practice) was simplified from three indicators to two, and the regression was re-estimated; (2) two additional control variables—“local governance preferences” and “proportion of shares held by the chairman”—were included alongside the original set of seven control variables, after which the regression was run again.

The results in Table 4 show that after the two robustness tests mentioned above, various management practices still significantly enhance productive performance outcomes, and all pass the significance test at least at the 5% level. The results are consistent with the benchmark regression, thus validating the robustness of the findings in this paper.

**Table 4:** Robustness test.

Variables	Productive Performance Outcomes Model 1 (OLS regression)				
	Method of calibration: (1) The independent variables are reduced to two indicators (2) Add two control variables (local governance preferences; proportion of shares held by the chairman)				
	Profitability	Institutional compliance	Technology dissemination	Employment generation	Community reputation
Planning practices	(1)0.315*** (2)0.262***				
Investment practices				(1)0.147* (2)0.161**	
Internal governance practices		(1)0.26*** (2)0.184***			
External collaboration practices			(1)0.51*** (2)0.401***		(1)0.405*** (2)0.364***
Monitoring practices					
Control variables (9 items)	Control	Control	Control	Control	Control
N	94	94	94	94	94
R^2^	(1)0.691 (2)0.684	(1)0.613 (2)0.635	(1)0.653 (2)0.614	(1)0.638 (2)0.668	(1)0.759 (2)0.762

***, ** and * represent significance levels of 1%, 5% and 10% respectively.

#### 6) Endogeneity tests.

To mitigate potential reverse causality between the dependent and independent variables, the key independent variable is instrumented using its one-period lag (mid-2025), denoted as L.Independent Variable. First-stage estimates indicate a strong association between the instrument and the contemporaneous independent variable. The exclusion restriction is deemed satisfied, as the lagged measure is unlikely to be influenced by current values of the dependent variable. As reported in Table 5, the second-stage estimates remain positive and statistically significant at the 5% level, supporting the baseline results after accounting for endogeneity.

**Table 5:** Results of the second stage regression of the instrumental variable method.

L.Independent Variable	Productive Performance Outcomes Model 1 (OLS regression)				
	Method of calibration: two-stage regression with instrument				
	Profitability	Institutional compliance	Technology dissemination	Employment generation	Community reputation
L.Planning practices	0.282***				
L.Investment practices				0.136**	
L.Internal governance practices		0.243***			
L.External collaboration practices			0.386***		0.352***
L.Monitoring practices					
Control variables (9 items)	Control	Control	Control	Control	Control
N	94	94	94	94	94
R^2^	0.676	0.656	0.589	0.655	0.715

***, ** and * represent significance levels of 1%, 5% and 10% respectively.

Table 6 presents Granger-causality tests incorporating five lags to assess whether past performance indicators predict future managerial practices. Only two pairwise relationships are significant at conventional levels: institutional compliance Granger-causes investment practices, and profitability Granger-causes internal-governance practices. These sporadic links do not coincide with the core explanatory variables in the baseline model and are insufficient to suggest systematic reverse causality. The general absence of bidirectional feedback thus strengthens the credibility of the main findings.

**Table 6:** Results of Granger-causality tests.

Pairing variables		F	P	Causality tests
Profitability	Internal governance practices	2.336	0.050**	One-way influence, with no two-way causation.
Internal governance practices	Profitability	1.255	0.292	
Institutional compliance	Investment practices	2.501	0.037**	One-way influence, with no two-way causation.
Investment practices	Institutional compliance	1.805	0.122	

***, ** and * represent significance levels of 1%, 5% and 10% respectively.

#### 7) Mediation effect test.

Given the absence of direct effects of monitoring practices on productive performance outcomes, this study further investigates whether monitoring exerts indirect influences through other mediating mechanisms: Planning practices, Investment practices, Internal governance practices, and External collaboration practices. To test these mediation pathways, we implemented the three-stage analytical framework (Models 2–4) outlined in Section 2.3. The results, detailed in Table 7, reveal significant indirect effects. The parallel mediation analysis revealed a significant indirect pathways:

**Table 7:** Mediating effects of monitoring practices.

Test Item	a-value	b-value	Indirect effect a × b-value	Direct effect c’-value	Test conclusion
Monitoring practices → Planning practices→Profitability	0.636 ***	0.351 ***	0.223 ***	−0.158 **	Mediator effects
Monitoring practices→Investment practices→Employment generation	0.563 ***	0.237 ***	0.134 **	0.057	Mediator effects
Monitoring practices→Internal governance practices→Institutional compliance	0.571 ***	0.293 ***	0.167 ***	−0.097	Mediator effects
Monitoring practices→External collaboration practices→Technology dissemination	0.505 ***	0.488 ***	0.247 ***	−0.095	Mediator effects
Monitoring practices→External collaboration practices→Community reputation	0.505 ***	0.389 ***	0.197 ***	−0.06	Mediator effects

***, ** and * represent significance levels of 1%, 5% and 10% respectively.

Positive mediation is achieved through monitoring practices. The significant path coefficients are a = 0.296 and b = −0.158, while the significant mediating effect is c′ = 0.351. The bootstrap 95% confidence interval for the indirect effect is [−0.142, −0.001], which excludes zero. The results show that the regression coefficient increased from 0.263 to 0.351 after adding the mediating variable Monitoring practices, and was significant at the 1% significance level. Monitoring practices exclusively enhance profitability by promoting improved planning, thereby acting as an institutional facilitator rather than a direct driver. This finding indicates that monitoring practices alone does not enhance productive performance outcomes; instead, a distinctive improvement path emerges only when monitoring is combined with other managerial practices such as planning practices etc..This is consistent with the results of the benchmark regression, indicating that monitoring practices play a partial mediating role in influencing performance outcomes.

#### 8) Influence path of management practices.

**Fig 7:**
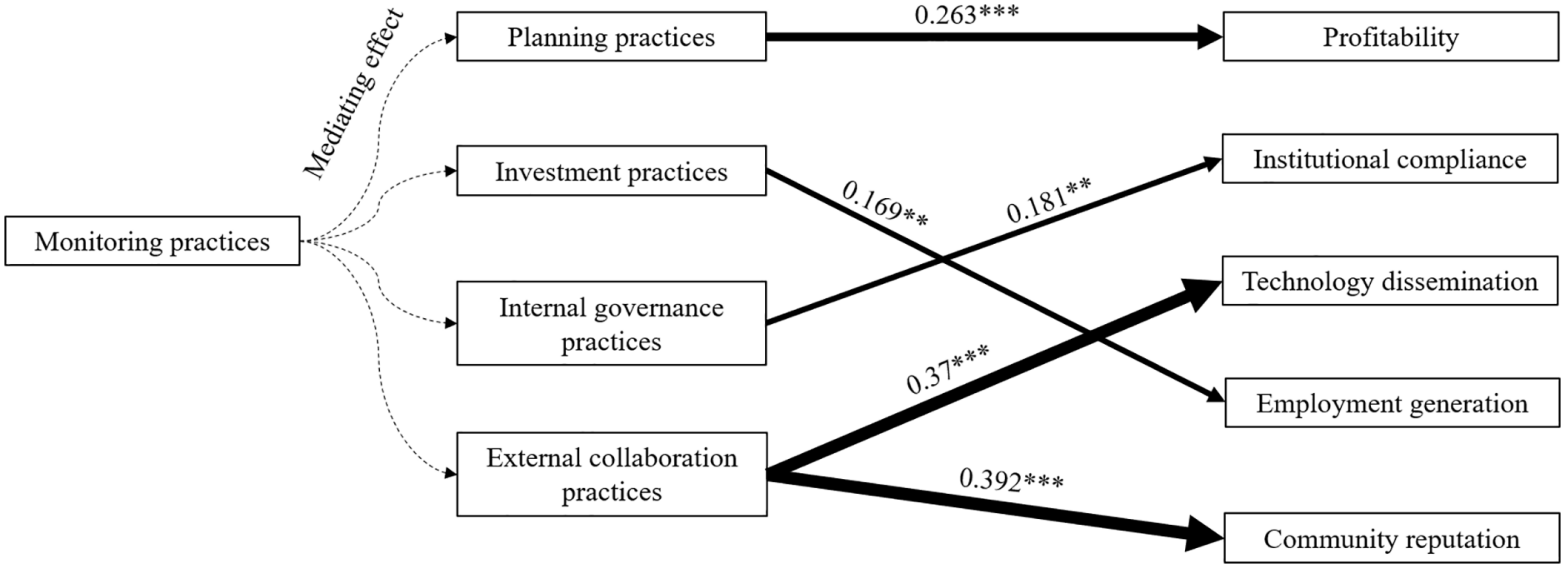
Influence path of management practices on performance outcomes. The α-value represents regression coefficient, the p-value represents significance levels.

The analysis demonstrates that distinct management practices differentially influence FPCs productive performance outcomes across multiple dimensions, as illustrated in Fig 7.

Planning practices significantly affect profitability (α = 0.263, p < 0.001). Strategic planning practices strengthen revenue streams.

Investment practices have a major impact on employment generation (α = 0.169, p < 0.05). Workforce training investments enhance procedural standardization, while capital infusion boosts production capacity, thereby increasing labor demand.

Internal governance practices mainly influence institutional compliance (α = 0.181, p < 0.005). Operational transparency and role specialization guarantee adherence.

External collaboration practices have a multidimensional impact on technology dissemination (α = 0.37, p < 0.01), community reputation (α = 0.392, p < 0.01). Cross-sector collaboration accelerates knowledge spillovers and facilitates reputation communication.

Monitoring practices have a latent role in enhancing productive performance outcomes via other managerial practices.

The above conclusions conclude that hypothesis H1 is valid, that is, different management practices of FPCs can produce different performance outcomes.

#### 9) Correlation test.

Building upon the baseline regression and mediation analysis outcomes, this study confirms that management practices critically determine cooperative productive performance. To further identify the most impactful behavioral indicators, we conducted a comprehensive correlation analysis.

**Fig 8:**
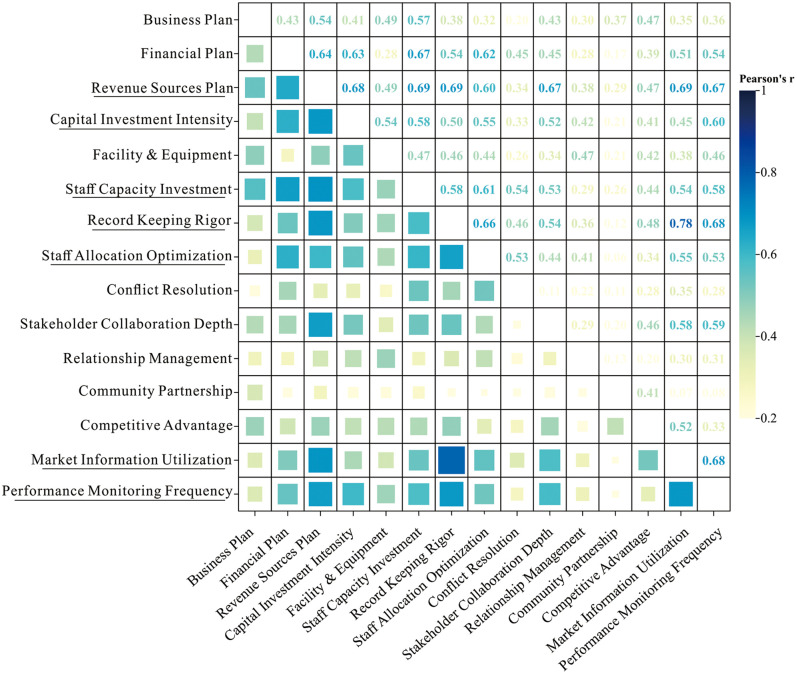
Inter group correlation of behavioral indicators. High correlation indicators are underlined.

Inter-behavior correlations (Fig 8), Seven operational indicators exhibited the most extensive influence networks across management dimensions (mean r = 0.48–0.56): Revenue source plan, Capital investment intensity, Staff capacity investment, Record-keeping rigor, Staff allocation optimization, Market intelligence utilization, Performance monitoring frequency. These indicators demonstrated significant pairwise correlations, functioning as linchpins of cooperative operational ecosystems.

**Fig 9:**
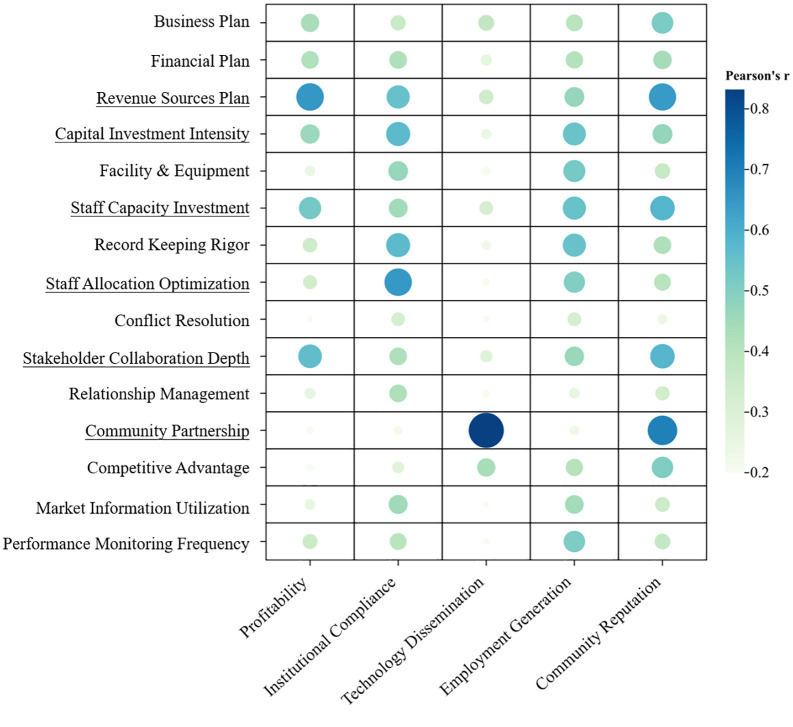
Correlation between behavioral indicators and performance outcomes. High correlation indicators are underlined.

Behavior-outcome correlations (Fig 9), Six behaviors emerged as critical drivers of high performance outcomes (mean r = 0.42–0.53): Revenue sources plan, Capital investment intensity, Staff capacity investment, Staff allocation optimization, Stakeholder collaboration depth, Community partnership. These behaviors are representative of those that constitute high productivity.

Based on the above, the four behaviors of Revenue sources plan, Capital investment intensity, Staff capacity investment and Staff allocation optimization are taken as the main control factors affecting the cooperative, as well as the operation and outcomes of the FPCs. Hence, during the developmental process, it is imperative for FPCs to meticulously assess their strengths and weaknesses, anticipate potential issues, and proactively implement corrective measures.

## Examples demonstrated

Effective high-level management practices within cooperative teams play a critical role in enhancing operational performance. A representative example is the Gaoshanyao FPC in Leye County, which specializes in edible oil processing and star anise cultivation. Leveraging the chairman’s background in infrastructure projects and wholesale trade, the cooperative benefited from substantial managerial expertise. Within its first year, the chairman established clear operational frameworks and regulations, mobilized partner engagement, and optimized internal workflows. These efforts contributed to consistently high standardized output metrics.

The management team actively monitored market trends and production data, strategically scaling output and aligning sales with periods of high demand. This approach generated nearly 200,000 yuan in profits by the second year, underscoring the cooperative’s strong profitability. Furthermore, the team secured loans and other forms of financing to alleviate working capital constraints, with subsequent investments in equipment and operations helping sustain financial performance. Long-term supply agreements with external manufacturers also provided a stable stream of revenue.

The cooperative’s operational success has positively influenced the local community, attracting 150 member households and strengthening its reputation. These developments have elevated its performance in areas such as technology dissemination and social recognition. Collectively, this case underscores how strategic management practices can drive superior operational outcomes.

## Conclusions and policy suggestions

### Conclusions

1) The management practices of farmer professional cooperatives (FPCs) can be classified into five distinct typologies: Comprehensive high-Efficiency type, Collaboration-deficient type, Monitoring-deficient type, Planning-deficient type, Holistically deficient type. Correspondingly, productive performance outcomes are categorized into five clusters: Profitable outreach high-efficiency type, Profitable insular high-efficiency type, Loss-making outreach high-efficiency type, Loss-making insular medium-efficiency type, Loss-making insular low-efficiency type. The analysis confirms that management practices critically determine cooperative performance typologies. Deficiencies in specific practices systematically degrade outcomes, with the absence of planning practices exerting the most substantial negative impact. Key findings include:

Planning-Deficient type FPCs exhibit 94% likelihood of clustering into Loss-Making Insular Low-Efficiency.
Collaboration-deficient type FPCs exhibit bifurcated outcomes, with 50% becoming loss-making insular medium-quality entities, as opposed to 23% that are profitable insular high-efficiency.
Monitoring-Deficient type FPCs 65% paradoxically achieve short-term profitability, but suffer long-term compliance risks.

2) The impact of management practices on cooperative productive performance varies across different dimensions, with distinct practice types influencing specific outcomes. Empirical evidence demonstrates that planning practices predominantly affect profitability outcomes, while investment practices exhibit a significant positive impact on employment generation. Therefore, FPCs that prioritize planning and investment—particularly value-added processing FPCs—should note that inadequate planning or investment may directly undermine profitability. Internal governance practices show strongest correlations with institutional compliance, whereas External collaboration practices primarily drive technology dissemination and community reputation. Consequently, FPCs that emphasize internal governance and external engagement—particularly those pursuing organic or green certifications—face significant operational risks when deficient in either domain. Inadequate internal controls or limited collaboration can lead to operational inefficiencies, hinder technological upgrading and knowledge continuity, dilute brand value, and ultimately jeopardize certification eligibility. Notably, monitoring practices demonstrate no direct causal relationship with productive performance outcomes. However, path analysis reveals their indirect contribution to performance outcomes via mediating effects exerted by other managerial practices. Service-oriented FPCs should therefore exercise particular diligence in monitoring practices. While monitoring deficiencies may not directly precipitate operational failure, they can lead to delayed adjustments in planning and investment behaviors, ultimately eroding profitability.
3) Among the behavioral indicators, the four factors—namely, Revenue sources plan, Capital investment intensity, Staff capacity investment and Staff allocation optimization—exert a significant influence on the operation and outcome of FPCs. These are the primary control factors that affect the development of FPCs.

### Policy suggestions

1) Efficient planning is fundamental to the sustainable development of FPCs (e.g., value-added processing FPCs), especially when it comes to the proactive planning of income sources.

Based on a comprehensive assessment of their own resource endowments, industrial infrastructure, and market demand patterns, FPCs should precisely define their development objectives and formulate well-structured business plans in advance. Moreover, in order to maintain competitiveness and adaptability in the face of a dynamic external environment, FPCs (e.g., Service-oriented FPCs) must establish a mechanism for the timely and dynamic adjustment of these plans. This adjustment process should be closely linked to market fluctuations and the actual development trajectory of the FPCs, ensuring that the business strategies remain relevant and effective in the context of constantly evolving circumstances.

2) To maintain competitiveness in increasingly saturated markets—particularly for organic and green-certified FPCs—developing distinctive branding is imperative. This entails crafting compelling brand narratives that communicate values of safety, health, and sustainability, while implementing comprehensive “Five-Unification” standardization across production processes: unified input supply, unified cultivation protocols, unified brand packaging, unified sales channels, and unified profit distribution. Such integration positions “certified organic and safe production” as the cornerstone of brand competitiveness.
3) Emphasize the investment in personnel training by ensuring that managers receive comprehensive training in various competencies. The government should integrate the training of agricultural management personnel into the annual work plan of the agricultural and rural affairs department. By developing long-term and systematic training programs, the continuity and stability of training efforts can be guaranteed. Utilize diverse training formats, including specialized lectures, case analysis, discussion and exchange sessions, on-site instruction, and online training. Additionally, conduct exchange activities to share management experiences and development models, thereby enhancing the overall quality of managers.

## Supporting information

S1 Data Management practices. (XLSX)

S2 Data Productive performance. (XLSX)

S3 Data Classification results-Management practices. (XLSX)

S4 Data Classification results-productive performance. (XLSX)

S5 Data The relationship. (XLSX)

S6 Data Statistical control variables. (XLSX)

S7 Data Robustness test data. (XLSX)

S8 Data Endogeneity test data. (XLSX)
